# Technology Empowers Emotions: How AR Technology Triggers Consumers’ Purchase and Spread Behavior Towards Intangible Cultural Heritage Brands

**DOI:** 10.3390/bs16010134

**Published:** 2026-01-17

**Authors:** Yi Sheng, Jiajia Zhao, Euitay Jung

**Affiliations:** 1 Department of Communication Design, College of Design, Graduate School of Hanyang University, Seoul 04763, Republic of Korea; sy0605@hanyang.ac.kr (Y.S.); zhaojia31531@hanyang.ac.kr (J.Z.); 2 Department of Communication Design, Hanyang University ERICA, Ansan 15588, Republic of Korea

**Keywords:** augmented reality technology, intangible cultural heritage, consumer emotion, cognition, purchase intention, brand communication

## Abstract

In recent years, the application of augmented reality digital technology in brands has transformed the way consumers interact with brands. This study focuses on the impact of augmented reality (AR) technology on consumption behavior and brand communication related to intangible cultural heritage products, integrating the TAM and UTAUT2 theories to construct a research model. This study employed a time–location sampling method, utilizing SPSS and AMOS software for data analysis based on valid questionnaires completed by 305 AR-experiencing consumers in Changsha City, Hunan Province. Results indicate that the presence and novelty of AR technology significantly and positively influence consumers’ attitudes toward using AR technology, which in turn affects their purchase intent, social media sharing behavior, and brand attitudes. The study confirms that emotional factors and consumer perceptions play a guiding and decisive role in the new consumption reality enabled by AR technology. These research findings have practical significance and value for ICH brand building and AR marketing, demonstrating that AR is an effective means to enhance the visibility and influence of the ICH brand. They inject new vitality into promoting more sustainable ICH protection and popularization, as well as the development of the digital creative industry.

## 1. Introduction

Intangible cultural heritage (ICH) refers to the non-material expressions of a community’s creativity, which are unique and distinct from those of other communities. These expressions encompass language, rituals, beliefs, literature, artistic works, and crafts (Lenzerini, 2011). ICH represents the cultural wealth utilized by our ancestors in daily life and passed down to the present day. Naturally developed and constantly changing throughout history, it reflects the special thoughts, traditions, and artistic tastes of different ethnic groups, keeping the true essence of their way of life. Due to its strong regional distinctiveness, the richness, scarcity, and recreational value of the area serve as key drivers for the development of tourism destinations. In recent years, ICH has increasingly been integrated into brand marketing strategies to attract local consumers. As the tangible form of ICH products, the ICH brand serves as the material carrier of traditional craftsmanship and a primary embodiment of ethnic, traditional, and regional cultures. Research indicates that building local brands through ICH not only enhances the cultural significance of the region but also integrates visitors with it. This approach ensures the transmission and preservation of knowledge while strengthening cultural foundations (Baksi & Sanyal, 2024)

However, in this information age of rapid technological advancement, industrial mass production has become the norm. In this era of rapid development, we are forgetting traditional crafts and ICH—those “time-consuming” practices. In some regions, ICH resources remain relatively unknown, and their cultural value is not widely recognized. Amidst the cultural shocks brought by the new media era, ICH must keep pace with the times and continuously innovate to stand out and gain visibility in this age of information overload. Currently, the landscape for ICH innovation is not optimistic, and ICH is facing the challenge of marketization in the information age (Yu, 2023). Low engagement and weak awareness of preservation pose numerous challenges to safeguarding these cultural heritage sites (K. Zhang & Liu, 2024). By the end of 2023, the Sustainable Development Goals (SDGs) had been in existence for eight years. Progress reports subsequently released on the United Nations official website indicated that the SDGs had made significant advances toward addressing fundamental needs such as housing, transportation, pollution, and waste management. However, the protection and promotion of cultural heritage had clearly not received sufficient attention (El Faouri & Sibley, 2024). Moreover, if cultural heritage protection policies evolve to meet contemporary needs while solely emphasizing conservation and neglecting sustainable utilization strategies, this protection-centered, regulation-focused model may ultimately prove unsustainable (Y. Chen et al., 2025). The aforementioned arguments reflect the developmental dilemmas and challenges currently facing ICH. However, these very dilemmas and challenges provide ample justification for the necessity of this research.

The continuous advancement of mobile and wearable devices capable of displaying digital content within physical environments, coupled with progress in 5G internet technology, has profoundly transformed how consumers interact with brands. Recently, consumer behavior has undergone rapid shifts, with an increasing number of people gravitating toward digital consumption and experiences (Shah & Murthi, 2021). Augmented reality (AR) technology is a media technology that merges virtual and real worlds, increasingly becoming a hot research area in brand design and communication. As one of these emerging technologies, it enables consumers to interact with brands in novel ways. AR offers immersive, interactive, and realistic experiences, meaning it can help businesses establish emotional connections with consumers (Pozharliev et al., 2022). AR technology can trigger a sense of intimacy between users and virtual objects. As research indicates, marketers can benefit from this perceived physical closeness—particularly in brand management (Rauschnabel et al., 2024). The advantage of AR technology lies in enabling consumers to interact with products through smart mobile devices, providing them with greater added value and delivering rich, authentic sensory experiences. Consequently, it has become a crucial tool for brands in promotion and marketing. Previous research indicates that the interactivity and virtual nature of AR experiences within brands enhance enjoyment, creating an immersive and pleasant shopping environment. AR not only fosters positive consumer experiences but also influences consumer behavior (Jiang & Lyu, 2024).

AR technology is overlaying digital information onto the real world in unprecedented ways, revolutionizing the tourism and cultural heritage sectors by creating deeply immersive experiences. On the one hand, AR enhances the interaction of users with educational items related to cultural heritage by offering them a view of real-world sites and artifacts. AR digitally overlays information, thus creating a contextualized visualization understanding that facilitates better comprehension (Boboc et al., 2022).

On the other hand, in the realm of smart tourism, AR technology enhances the perception of time and space by seamlessly integrating virtual information into tangible objects and spaces, and facilitating interaction between multimedia information and the real world (Carrion & Levinson, 2012).

Currently, renowned companies and brands in the market are leveraging extensive innovative technologies to enhance consumer shopping experiences, with AR technology emerging as a pivotal step. The popularity of AR continues to rise, driven by the rise of virtual try-on features from brands like Nike and Gucci, alongside AR advertising experiences and promotional campaigns from prominent food and beverage brands such as Coca-Cola, Burger King, and Red Bull. Additionally, online retailers like IKEA and Wayfair offer unique shopping solutions. Consumers can select furniture and use their smartphone cameras to project products directly into real-life settings.

Previous studies have extensively explored consumer brand experiences through the lens of AR (Butt et al., 2024; Ksenija et al., 2024). However, by synthesizing prior research, it is evident that many scholars have examined the impact of AR technology on consumer brand experiences and brand recognition within food and furniture brands (Arce-Lopera et al., 2021; Rauschnabel et al., 2019). Nevertheless, cases citing AR experiences within ICH brands remain scarce, and related research appears somewhat limited. This scarcity presents a favorable entry point for the present study. This study focuses on consumer experiences with ICH brands utilizing AR technology and the psychological impact of these experiences on ICH brand communication and consumers. While some researchers have integrated ICH, education, and technology to explore the application and influence mechanisms of AR technology in ICH tourism (Li et al., 2022), others have examined the relationship between customers and brand experiences based on VR and AR from the perspective of brand authenticity (Zeng et al., 2023).

However, there remains a scarcity of research on ICH brands employing AR technology and its effects on brand communication and consumer psychology. In previous research on ICH and digital technologies, scholars have noted that cultural awareness and general learning outcomes (GLO) influence users’ perceived usefulness and perceived ease of use. Perceived usefulness and perceived ease of use exert a positive influence on attitude toward using, which in turn positively affect the intention to use (Kang et al., 2023). This study did not clarify or confirm which variables, beyond perceived usefulness and perceived ease of use, could influence consumer attitudes and behaviors. In case studies utilizing AR technology for virtual ICH scene presentation, researchers emphasize that the interaction between AR design and real-world intangible cultural heritage cases is crucial, as it deepens understanding of local cultural characteristics (Shih et al., 2021).

Moreover, researchers based on the S-O-R theory explored the extent to which AR technology affects the willingness to purchase handicrafts in terms of vividness, information quality, and interactivity (Hu et al., 2025). In addition to the attributes and characteristics of AR technology previously discussed, it is imperative to examine the influence of additional attributes and characteristics on consumer behavior. Furthermore, it is crucial to explore the role of emotional factors in triggering these behaviors. These inquiries remain understudied and unproven. Therefore, the following research questions will be addressed:

RQ1. What are the core attributes or characteristics of AR? What is its relationship with consumers’ purchase intentions and brand attitudes toward ICH brands?

RQ2. What is the mechanism through which AR influences consumer attitudes and behaviors based on its core characteristics within ICH brands?

RQ3. Which emotional factors influence consumer attitudes and behaviors through AR’s technological attributes within ICH brands?

RQ4. How do emotional factors influence consumer attitudes and behaviors through AR’s technological attributes within ICH brands?

To address these questions, a comprehensive research framework must be established based on prior studies to fully elucidate how AR attributes impact consumer attitudes and purchase intentions toward ICH brands within emotional experiences.

## 2. Literature Review

### 2.1. Augmented Reality and Its Attributes

According to Azuma’s (1997) definition at the University of North Carolina, AR technology encompasses the following three aspects: AR technology integrates virtual objects with reality, enables real-time interaction, and constructs three-dimensional worlds. Its underlying principle can be summarized as follows: using computer technology to simulate and overlay entities—such as visual, auditory, olfactory, or tactile information—that are difficult to perceive within a specific time and space in the real world. This virtual information is then applied to the physical world, becoming perceptible to human senses and delivering sensory experiences that transcend reality. Therefore, AR is a technology similar to virtual reality (VR) that aims to enhance or enrich the viewer’s experience (Azuma, 1997). It possesses unique media capabilities, enabling real-time interaction while blending the real and virtual worlds. Distinct from existing virtual reality technologies, it creates a “mixed reality” format where the surrounding environment remains real, yet the depicted and displayed environment of objects is virtual (Cho & Schwarz, 2012).

AR enhances people’s perception and experience of virtual spaces that blend the real and virtual worlds. Previous studies have indicated that AR can create a high degree of interactivity (McLean & Wilson, 2019). Interactivity has been recognized as a key concept for successful online and mobile marketing and communication. Scholars have proposed definitions of interactivity from four perspectives: the process of information exchange, technological characteristics, users’ perceptions of experiential technologies, and a combination of these three dimensions (McMillan & Hwang, 2002). Fundamentally, computer-generated artificial worlds confine user interaction and attention within computational spaces. In virtual environments, users must detach from their physical surroundings to achieve full immersion within the synthetic world. Particularly when users must decide when to synchronize interactions with the real world, divided attention and disjointed interactions may produce unexpected negative impacts on task performance and safety (Kim & Dey, 2010).

Beyond interactivity, presence is another core concept in AR. Presence is defined as “a state of consciousness, a (psychological) sensation of being within a virtual environment.” (Slater & Wilbur, 1997). In previous studies, presence has been described as a multidimensional concept encompassing self-presence, social presence, and physical presence (Biocca, 1999). Unlike VR, the presence generated by AR technology originates from spatial and physical dimensions (Aitamurto et al., 2022). It embodies the diverse “immersive” perceptions experienced by users within virtual environments. This concept has been applied to understand visitor responses within immersive tourism frameworks, serving as a crucial theoretical foundation for studying user or visitor experiences and reactions in virtual settings (J. Chen et al., 2023). When an individual experiences a subjective sense of presence despite not physically occupying an artificial environment, presence is generated (Draper et al., 1998). In consumer settings, presence primarily stems from consumers’ experiences within virtual environments, the 3D presentation of products, and the construction of virtual spaces. It enhances consumer perceptions through its various dimensions (Daassi & Debbabi, 2021). The theory of presence posits that consumers can perceive physical existence within AR experiences without entering a real-world space. Creating the illusion of virtual objects within the real physical environment generates a sensation that transcends conventional shopping settings.

Beyond the aforementioned AR attributes, novelty is also a crucial aspect of the AR experience. According to Smith et al. (2007), novelty is defined as the originality, creativity, flexibility, and artistic value demonstrated by designers during the creative process, collectively forming the core characteristics of AR novelty. The novelty embodied in AR visual content reflects designers’ original thinking. It showcases the boundaries of their design capabilities—specifically, how to create captivating visual imagery in an environment free from physical and tangible constraints. Uniqueness and novelty in AR captivate consumers through unprecedented visual and interactive experiences, sparking their curiosity and desire to explore. For consumers, the creativity of AR visual content fuels their pursuit of novelty and personalized experiences, fulfilling their need to express themselves and embrace individuality within virtual environments. Novelty is also evaluated as the degree of being “new, unique, and different,” a fundamental characteristic of any innovation. The perceived novelty of an idea or product significantly influences subsequent reactions (Rogers et al., 2014). The novelty perceived during an experience reflects people’s perception of a technology as novel, engaging, and distinctly different from other technologies they use or understand. The novel stimuli within this technology evoke a sense of unfamiliarity and spark interest in its content. Increasing interest in these novel stimuli activates arousal states, prompting individuals to seek more information about them (Magni et al., 2010).

### 2.2. AR and Brand Marketing

AR has also been applied to brand marketing, a practice that leverages AR technology to support corporate marketing efforts. Users can interact with products through AR-enabled mobile devices, smart glasses, or headsets. The methods for utilizing AR in marketing are diverse, primarily categorized into virtual try-ons, enhanced brand materials, virtual home visualizations, augmented showrooms, and experiential marketing. Currently, the advertising industry is a key area for AR exploration. For instance, users can scan products via their phones or projectors to trigger AR devices, displaying interactive virtual styling options like virtual try-ons on screens. This technology delivers sensory experiences that transcend reality. Common AR marketing objectives include brand building, inspiration, persuasion, and recall (Rauschnabel et al., 2022). Recently, companies have been strategically exploring experiential marketing across multiple dimensions to integrate experiences with their products or services and create added value, particularly by adopting a customer-centric approach. Promoting experiential consumption combined with AR technology can be considered an essential strategy for generating corporate profits. Previous research findings indicate a positive correlation between AR usage and the development of brand affection (Afonso & Hipólito, 2022).

Augmented reality (AR) and virtual reality (VR) technologies are transforming how fashion brands engage with consumers, blurring the lines between the physical and digital worlds. The fashion industry extends far beyond clothing: it encompasses experiences, creativity, and self-expression. Adidas integrates augmented reality into its in-store displays. Customers can use AR-enabled mirrors to visualize shoes on their feet, access product information, and even watch product videos. This application of augmented reality technology elevates the in-store experience, enhances customer interaction with products, and provides shoppers with valuable information (Madhu, 2024). These immersive technologies not only improve the shopping experience but are also transforming design, marketing, and retail operations. Reports indicate the AR shopping market is projected to grow to $3.4 billion in 2023 and reach $11.6 billion by 2028 (Sayed, 2019).

Furthermore, AR technology offers a convenient opportunity to transform the customer experience within retail environments. For instance, AR can act as a link that connects physical stores with online shops, combining the advantages of both. By creating virtual environments integrated with reality, AR can heighten consumers’ curiosity about brands, thereby encouraging them to invest time and effort in exploring and engaging with them. Previous research findings indicate that AR enhances brand recall and recognition by creating immersive environments. AR enables participants to recall more details and information about products. The impact of carefully designed AR experiences on brand recall and recognition opens up possibilities for new technologies in retail settings (Arce-Lopera et al., 2021). AR possesses strategic capabilities to attract consumers and enhance brand perception, brand competitiveness, and social value by improving user consumption and usage experiences, thereby strengthening individuals’ attachment to brands incorporating AR technology (Arya et al., 2025).

As an interactive technology, AR’s development has not been achieved overnight but has undergone extensive technological accumulation over time (as shown in Table 1). AR has transitioned from the laboratory to everyday life, gradually evolving into a lightweight, portable digital medium. Its role in brand marketing has shifted from being a mere attention-grabbing gimmick to becoming a core tool for enhancing user experience and driving business growth.

### 2.3. ICH Brand and AR

Cultural heritage represents the immense legacy of human history, categorized into tangible cultural heritage and intangible cultural heritage (Hua, 2010). Tangible Cultural Heritage (TCH) encompasses the physical vestiges of human activities, creations, developments, and achievements, such as towns, palaces, villages, temples, and tombs. In contrast, intangible cultural heritage (ICH) represents knowledge and skills that exist in non-material forms. ICH includes the performing arts, local knowledge, traditional skills, and languages. ICH possesses a distinct identity that conveys the sense of national identity, uniqueness, and diversity embedded within its materials and textures (Sawagvudcharee, 2024).

Against the backdrop of cultural homogenization brought about by economic globalization, ICH has become a vital component in safeguarding cultural diversity and developing the tourism economy (Timothy, 2014). Recently, the role of ICH in economic development has garnered increasing attention, with many cities leveraging it as a renowned tourism resource to promote sustainable urban development (Schildenfeld & Odak Krasić, 2020). On one hand, ICH reflects diverse local narratives and serves as a vital element in destination branding (Ryan, 2015). Local administrators often interpret the outcomes of branding as products of cultural and economic integration, thereby embedding the branding process within local social spaces and fully leveraging the distinctive expressions of local culture (Kavaratzis & Hatch, 2013).

On the other hand, branding serves as a model for safeguarding ICH and revitalizing cultural tourism (Qiu et al., 2020). Relevant studies suggest that ICH that is adequately protected, restored, promoted, and capitalized upon can support the construction and utilization of national brands, enhance national soft power, and strengthen the competitiveness of the national tourism industry (Vegheș, 2022). UNESCO branding research indicates that World Heritage site branding plays a crucial role in raising visitor awareness and promoting specific cultures. It can increase tourism demand and visitor patterns in historic centers while enhancing destination tourism quality (Hassan & Rahman, 2015).

Throughout China’s long cultural history, a vast accumulation of precious, rich, and splendid tangible and intangible cultural heritage has been preserved. As an exceptionally important form of cultural heritage, ICH is regarded as the cultural DNA embodying the unique diversity of China’s traditional culture. However, against the backdrop of rapid modernization and accelerated urban–rural integration, the survival environment of intangible cultural heritage faces numerous pressing practical challenges that require urgent attention (Xu, 2018). Advances in digital technologies such as AR offer promising avenues for safeguarding and transmitting intangible cultural heritage within the arts and culture sectors, signaling that heritage preservation is entering a dynamic and evolving frontier phase. Digital technologies are playing an increasingly vital role in the protection and dissemination of intangible cultural heritage. Precedents already exist for utilizing AR technology to preserve Chinese murals, such as the digitization efforts undertaken for the Dunhuang Grottoes murals in China (Zheng, 2024).

The use of interactive technologies such as AR has brought significant innovation to cultural heritage preservation and promotion. Currently, learning and tutoring appear to be the most prominent research areas for AR applications (Akçayır & Akçayır, 2017; Pietro et al., 2018). Following its positive impact in education (e.g., learning motivation, memory retention), the technology has expanded into marketing and alternative tourism (Chiao et al., 2018; Ramos et al., 2016). Often integrated with other mobile technologies like cameras and GPS tracking, it enhances travel experiences while offering innovative ways for people to explore new places and cultures.

However, branding as a form of modern cultural expression plays a vital role in the protection and transmission of ICH. Integrating ICH brands with immersive technologies like AR becomes crucial for promoting brand services, reinforcing brand image, advancing brand marketing, and enhancing brand product quality. Previous research indicates that ICH protection can yield better outcomes within brand strategies. Furthermore, leveraging new technologies to elevate ICH consumers’ recognition of ICH brand value helps drive their purchasing behavior toward the brand (J. Zhang et al., 2024).

## 3. Conceptual Model and Hypotheses

### 3.1. Technology Acceptance Model and Related Theories

AR possesses distinct attributes and functionalities that set it apart from other e-commerce technologies, rendering existing theories inadequate for fully explaining the influence of AR technology on the relationship between users and ICH brands within AR contexts. Researchers studying AR must focus on identifying which AR characteristics and functionalities elicit specific consumer responses in ICH brands utilizing AR technology. This study will enable a theoretical and practical understanding of how to attract consumers and sustain and develop their interactions with ICH brands. This study proposes a comprehensive theoretical framework grounded in the Technology Acceptance Model (TAM) (Venkatesh & Davis, 2000) and the Unified Theory of Acceptance and Use of Technology 2 (UTAUT2) (Venkatesh et al., 2003). This framework provides a practical conceptual foundation for constructing research on the impact and role of introducing AR experiences in ICH brands on consumers’ purchase intentions and brand attitudes.

The TAM model aims to predict behavior and intention based on attitudes and beliefs, with this theoretical framework grounded in the Theory of Reasoned Action (Goebert & Greenhalgh, 2020). Behavior is determined by behavioral intention, which is influenced by attitude. Conversely, attitude is formed based on various beliefs. For example, certain beliefs (such as perceived usefulness or simplicity) may lead to positive attitudes, while others (such as perceived tediousness or costliness) may result in negative attitudes. TAM focuses on two key beliefs: perceived ease of use and perceived usefulness. First, perceived ease of use is crucial because a technology requiring significant effort and being challenging to learn is unlikely to be adopted. Second, perceived usefulness is equally important because a technology offering no advantages over traditional methods is unlikely to be adopted. Previous researchers have applied the TAM model to investigate how AR interacts with consumers in marketing contexts and influences their purchasing decision-making processes (Eru et al., 2022).

The UTAUT2 model is a unified model of technology acceptance and use, developed from the original UTAUT model. The UTAUT model identifies four core variables: performance expectancy, effort expectancy, social influence, and facilitative conditions. The rise of consumer technology necessitated extending the UTAUT model to consumer contexts. Consequently, Venkatesh and his team further developed the UTAUT2 model in 2012, building upon the UTAUT framework by incorporating three additional variables: hedonic motivation, price value, and habit. Compared to UTAUT, UTAUT2 better explains consumer acceptance and usage of new technologies across different contexts, demonstrating significantly higher predictive power. It accounts for 74% of the variance in consumer behavioral intentions and 52% of the variance in consumer technology usage (Venkatesh et al., 2012). The UTAUT2 model has been applied to research involving various technology types, such as e-commerce platforms, social networking sites, and wearable technologies. It focuses on diverse user groups—including consumers, citizens, and employees—and the various tasks they perform, such as consumption, teaching, learning, accessing electronic health records, and travel.

### 3.2. Interactivity

In AR properties, interactivity refers to the user’s ability to control what they see in both the real and virtual worlds (McLean & Wilson, 2019). Interactivity arises when consumers choose to engage with AR content. As a defining characteristic of media and intelligent digital technologies, interactivity directly influences consumer experiences. AR can create more interactive and enjoyable shopping experiences, thereby enhancing consumer engagement (Pantano & Servidio, 2012). Previous researchers experimentally verified the influence of interactivity within AR attributes on consumer responses and perceptions (McLean & Wilson, 2019). Their findings indicate that, moderated by immersion, interactivity within AR attributes positively influences consumers’ perceptions of practicality and entertainment value. The realization of perceived benefits within AR experiences fosters positive attitudes toward AR applications.

Furthermore, a positive relationship exists between AR interactivity and consumer brand engagement, which in turn positively impacts consumer experience satisfaction and future brand intention. Researchers have also examined AR from the perspective of its perceptual interactivity dimensions. Findings indicate that controllability and enjoyment within perceptual interactivity influence consumers’ mental imagery, which in turn affects their attitudes toward the product and behavioral intentions. However, the relationship between perceptual interactivity and mental imagery varies depending on the individual’s level of engagement (Park & Yoo, 2020).

By extending prior AR interactivity research into the integrated TAM and UTAUT2 framework, AR attributes within ICH brands may serve as triggers for consumer value perceptions (perceived usefulness, perceived usability, and hedonic motivation). Therefore, the following hypotheses are proposed:

**H1a.**  *AR interactivity in ICH brands positively influences consumers’ perceived usefulness.*

**H1b.**  *AR interactivity in ICH brands positively influences consumers’ perceived ease of use.*

**H1c.**  *AR interactivity in ICH brands positively influences consumers’ hedonic motivation.*

### 3.3. Presence

Presence is one of the key concepts and attributes in AR virtual environments, referring to the various “immersive” sensations experienced by users within the virtual environment (Lyu et al., 2021; Zhu et al., 2024). The overarching concept of presence has also been employed to explain and describe users’ feelings and responses within immersive experience frameworks (Song & Lu, 2024; Wu & Lai, 2023). Presence stems from the sense of existence generated by the interaction between the real world and the virtual world, enabling users to set aside their doubts and believe they are situated within the virtual environment.

In TAM theory, prior researchers have argued that when a technology is difficult to use, its perceived benefits diminish, thereby reducing customers’ ability and willingness to adopt it (Blut & Wang, 2020). Previous studies have examined the mediating role of perceived ease of use in the relationship between presence and customer responses (Jung et al., 2018). Similar to perceived ease of use, perceived usefulness is a crucial variable in TAM. It refers to an individual’s belief that a specific technology will help them perform tasks or work more effectively (Chiao et al., 2018). Within TAM, perceived ease of use serves as a prerequisite for perceived usefulness, shaping users’ intentions and behaviors toward a particular technology (Jung et al., 2018).

Hedonic motivation refers to the degree of pleasure and perceived enjoyment individuals derive from using new technologies. It has been incorporated as a model variable in UTAUT2. Perceived enjoyment is crucial in the AR/VR domain, with prior research demonstrating its mediating role between presence and customer reactions (Chung et al., 2015). Their findings indicate that while presence influences perceived enjoyment, perceived enjoyment does not independently elicit purchase intention for online travel products. Consequently, research on the role of perceived enjoyment remains controversial. Integrating the above studies, the following hypotheses are proposed:

**H2a.**  *AR presence in ICH brands positively influences consumers’ perceived usefulness.*

**H2b.**  *AR presence in ICH brands positively influences consumers’ perceived ease of use.*

**H2c.**  *AR presence in ICH brands positively influences consumers’ hedonic motivation.*

### 3.4. Novelty

Previous research findings indicate that vividness and novelty are considered the most prominent attributes of AR, which may stimulate consumers’ relevant responses to brands. The novelty of AR is defined as “the novel, unique, and personalized content or stimuli experienced each time through an AR display” (McLean & Wilson, 2019). In previous research, Fang (2019) argued that personalized innovation features stimulate consumers’ curiosity, prompting them to further search for new information. Additionally, Rauschnabel et al. (2019) explained that AR applications with unique attributes—such as novelty, vividness, and quality—can evoke both functional and entertaining experiences for users. Previous researchers have found that the persuasive effect of AR advertising stems from the activation of consumer curiosity and that its effectiveness and novelty are constrained by consumers’ familiarity with AR technology (Yang et al., 2020).

Some researchers also contend that in video advertisements utilizing AR technology, novelty is the most crucial creative dimension. It correlates not only with short-term brand information recall but also closely relates to long-term brand information recall, advertising attitudes, and brand attitudes. Individuals’ prior brand attitudes influence their perceptions of advertising creativity for familiar brands. However, for unfamiliar brands, people lack preexisting attitudes, so their perceptions of advertising creativity are primarily shaped by how they evaluate the advertisements themselves (Feng & Xie, 2019). We propose the following hypotheses based on the above research findings:

**H3a.**  *AR novelty in ICH brands positively influences consumers’ perceived usefulness.*

**H3b.**  *AR novelty in ICH brands positively influences consumers’ perceived ease of use.*

**H3c.**  *AR novelty in ICH brands positively influences consumers’ hedonic motivation.*

### 3.5. Perceived Usefulness, Perceived Ease of Use, Hedonic Motivation

Perceived usefulness, perceived ease of use, and hedonic motivation are crucial value perception factors in TAM and UTAUT2. According to Davis (1989), users’ perceptions of a new technology’s usefulness and ease of use positively influence their behavioral attitudes and willingness to adopt it. Szajna (1996) found that perceived usefulness plays a dominant role in users’ adoption intentions. Furthermore, Bazelais et al. (2018) revealed that perceived usefulness and perceived ease of use are the primary motivators for users to adopt and use new technologies. These variables influence attitudes toward using AR technology and serve as key indicators for measuring user satisfaction with AR technology usage.

The pleasure consumers perceive is a significant outcome of their shopping activities (Babin et al., 1994). Although the utilitarian value of AR typically outweighs its hedonic value, pleasure remains crucial in consumers’ behavioral intentions to adopt AR for shopping and enhancing brand experiences (Schreuder et al., 2024). Furthermore, the fun factor of mobile shopping platforms can encourage consumer adoption of the technology and foster positive attitudes toward using AR (Khashan et al., 2023). We propose the following hypotheses based on the above research findings:

**H4.**  *Consumer-perceived usefulness of the ICH brand positively influences attitude toward using.*

**H5.**  *Consumer-perceived ease of use of the ICH brand positively influences attitude toward using.*

**H6.**  *Consumer-perceived hedonic motivation toward the ICH brand positively influences attitude toward using.*

### 3.6. Attitude Toward Using, Purchase Intention, Social Media Sharing, and Brand Attitude

Attitude toward using refers to a user’s positive or negative evaluation of a particular technology (Davis, 1989). The feasibility of this technology depends on users’ attitudes toward it. The most important factor motivating users to adopt a mobile learning system is their attitude toward this technology (Yadegaridehkordi & Iahad, 2012). Behavioral intention to use is a direct determinant of user adoption of a system. In contrast, actual use serves as an indicator for predicting actual user utilization rates based on user preferences. Li et al. (2022) indicate that behavioral intention to use positively influences actual use of the system. However, users’ attitudes toward using the mobile learning system directly affect their behavioral intention to use it (Al-rahmi et al., 2021).

AR is considered a valuable tool for delivering brand messages, not only helping consumers understand product concepts but also stimulating their exploratory behavior, which directly influences their purchase intention. Additionally, when using mobile AR technology, consumers with varying degrees of personalization or fashion innovation preferences exhibit differing purchase intentions toward brand products (Wang et al., 2022).

AR facilitates interaction and connection among consumers through designated applications. These applications offer a variety of functionalities, including the ability to follow or unfollow other users, send direct messages, and extend invitations to contacts from existing social networks. This interaction and connection enable the development of extensive online communities. Within this network, the broader the scope of consumer interaction and communication, the greater the likelihood of understanding each other’s challenges and difficulties with products. This peer-to-peer engagement, in turn, fosters deeper connections among consumers, thereby strengthening their emotional attachment and trust in the product (Aburub, 2023).

Although emotional responses can directly or indirectly enhance consumers’ purchase intent and word-of-mouth sharing intent through product or brand attitudes, AR itself cannot guarantee the generation of more positive emotional responses (Zanger et al., 2022). Previous research findings indicate that brand image significantly influences consumer perceived quality. In other words, when consumers hold a favorable image of a brand, they perceive both the product and non-product attributes of that brand as higher quality. Consumers’ positive perceptions or attitudes toward a brand, along with their psychological associations with it, make them more receptive to the brand’s new services, such as AR applications (Jiang & Lyu, 2024). Therefore, based on the above research, the following hypotheses are proposed:

**H7.**  *Consumers’ attitudes toward AR use positively influence purchase intention for ICH brands.*

**H8.**  *Consumers’ attitudes toward AR use positively influence social media sharing for ICH brands.*

**H9.**  *Consumers’ attitudes toward AR use positively influence brand attitude for ICH brands.*

**H10.**  *Consumers’ social media sharing positively influences purchase intention for ICH brands.*

**H11.**  *Consumers’ social media sharing of ICH brands positively influences brand attitude.*

**Figure 1:**
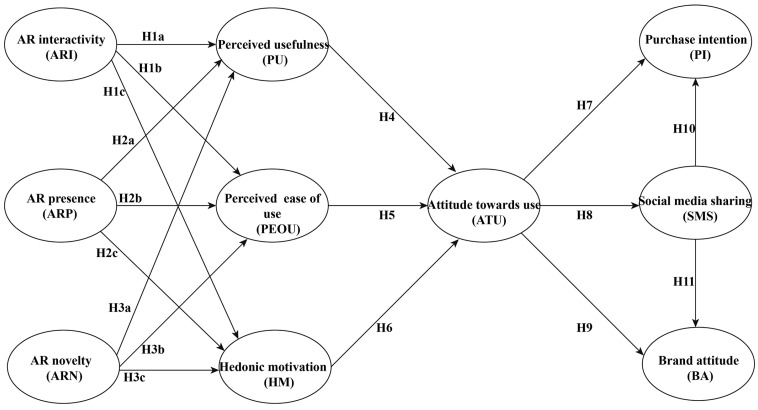
Research model. A conceptual model of the impact of ICH brand AR experiences on purchase intention. Source: The figure was created by the author based on assumptions.

Based on the aforementioned research hypotheses, the IBM SPSS Amos 26.0 software was employed to meticulously construct the research model examining the relationship between consumers’ AR experiences and purchase intention for ICH brands. The specific research model is illustrated in Figure 1.

## 4. Research Methods

### 4.1. Research Area and Objects

China is one of the world’s oldest nations with a rich and diverse ICH resource. Having acceded to the UNESCO Convention for the Safeguarding of the Intangible Cultural Heritage in 2004, the latest official statistics as of 2022 indicate that China boasts 3610 nationally designated ICH items. Additionally, China holds 44 entries on UNESCO’s ICH lists, ranking first globally. Geographically, the distinct population and urban distribution patterns between eastern and western regions significantly influence the spatial distribution of China’s ICH. Overall, it exhibits a pattern of greater concentration in the east and south, and lesser in the west and north (Z. Zhang et al., 2024). Hunan Province, located in south-central China, is a region rich in intangible cultural heritage brands. Situated between 24.7° N and 30.1° N latitude and 108.8° E and 114.2° E longitude, the area boasts abundant intangible cultural heritage resources. As of August 2021, Hunan had 137 items inscribed on China’s ICH list (Yuan et al., 2022). These ICH brands are predominantly concentrated in Changsha, the capital of Hunan Province, and its surrounding areas. As one of China’s new first-tier cities, Changsha boasts a vast consumer base for branded goods.

Furthermore, Changsha’s ICH brands demonstrate outstanding performance in branding and marketization, as shown in Table 2.

These ICH brands exhibit distinct regional characteristics, primarily clustered in historic cultural districts (e.g., Taiping Street, Pozhi Street, Beizheng Street) and traditional commercial hubs (e.g., May 1st Square, Huangxing Road Pedestrian Street). The types of ICH brands predominantly encompass traditional snack production, traditional Chinese medicine preparation, and noodle-making techniques.

**Figure 2:**
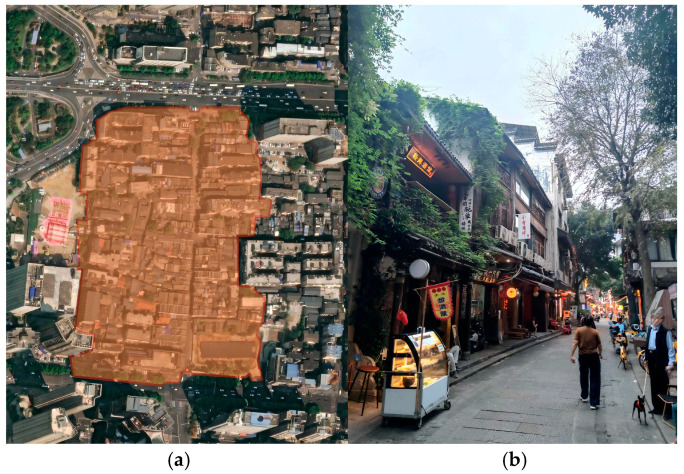
(**a**) Satellite map of Changsha Taiping Street; the area within the red frame is Taiping Street in Changsha; (**b**) real scene of Changsha Taiping Street. Source: (**a**) Image © 2025 Google Earth, Airbus; (**b**) this photo was taken by the author.

In recent years, the Changsha municipal government and surrounding universities have been actively promoting the digital preservation and innovation of ICH. Therefore, this study focuses on the ICH brand in Changsha, Hunan, selecting Taiping Street in Changsha City, Hunan Province, China as the AR display and experience area for the ICH brand. (As shown in Figure 2) Located in Tianxin District, Changsha, Taiping Street stretches 375 m from Wuyi Avenue in the east to Jiefang West Road in the west. With a history spanning two millennia, it stands as one of Changsha’s best-preserved ancient streets. Originally established during the Warring States period, it flourished as a commercial hub during the Qing Dynasty. To this day, it retains its distinctive “fishbone-shaped” alley layout, serving as a vibrant convergence of culture, tourism, cuisine, and commerce. Taiping Street, a cultural landmark of Changsha, is gradually emerging as a key testing ground for AR technology to empower the consumption of ICH brands.

### 4.2. Methods and Data Collection

This study employed an empirical approach. Based on a literature review, field research, and expert consultation, a preliminary scale measuring consumer experience and purchase intention toward Changsha ICH brands utilizing AR technology was developed. Subsequently, to ensure the scientific rigor and accuracy of the questionnaire survey, 30 pre-tests were distributed to experts and respondents. Based on the initial feedback received, items concerning brand attitude and presence within the scale were revised to eliminate duplicative questions—the final questionnaire comprised two sections. The first section included demographic information such as respondents’ age, gender, and place of residence. The second section measured and compiled data on consumer perceived value and purchase intent, as shown in Table 3. All items were adapted from previous studies. The final questionnaire comprised 42 items, all of which were translated into both Chinese and English. These items were reviewed and approved by two experts in the field. Ultimately, a five-point Likert scale (−2 = Strongly Disagree, −1 = Disagree, 0 = Neutral, 1 = Agree, 2 = Strongly Agree) was adopted for measurement and statistical analysis.

Subsequently, the questionnaires were collected and subjected to outlier detection based on response duration and consistency criteria, thereby excluding invalid responses. Finally, data analysis was conducted using IBM AMOS 26.0 and IBM SPSS 27.0 to perform confirmatory factor analysis (CFA), structural equation modeling (SEM), and hypothesis testing. Based on the analytical findings, research conclusions and recommendations were formulated.

### 4.3. Sampling

This study employed a time–location sampling method. To minimize variations arising from cultural cognitive differences and varying levels of technological acceptance—and considering that Taiping Street in Changsha possesses abundant ICH brand resources and a high pedestrian flow—the questionnaire survey and AR visual experience were conducted within Taiping Street, Changsha. The survey was carried out from 10:00 a.m. to 8:00 p.m., targeting individuals who appeared randomly during this period for recruitment and participation. However, discrepancies in the division and selection of sampling time slots may have introduced participation bias. Recruitment was conducted through proactive engagement and communication, as well as by posting advertisements. The questionnaires were distributed in the Taiping Street area of Changsha, Hunan Province, China, between 22 April and 10 May 2025. The target respondents were consumers residing in Changsha, Hunan Province, and its surrounding areas. Participation in the survey was conducted on a voluntary and anonymous basis. To ensure experimental safety and data validity, participants were required to meet the following criteria: no known color vision deficiency (color blindness/color weakness), no history of vertigo, photosensitive epilepsy, or related visual perception disorders, and no other physical health conditions that could potentially impact the AR experience.

**Figure 3:**
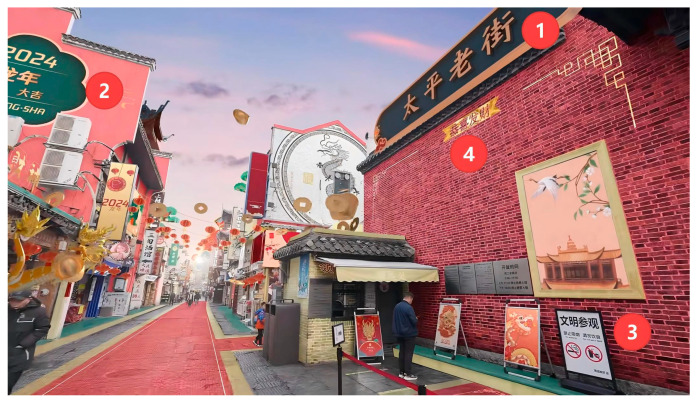
AR visual presentation of Changsha’s ICH brand. ① Taiping Old Street ② Auspicious Year of the Dragon 2024 ③ Please visit respectfully ④ Wishing you wealth and prosperity The images were composed using Adobe After Effects CC 2021 for dynamic element compositing and 3D modeling using Cinema 4D R21. Source: Your Changsha, My Changsha Seems Different © Mai A Zhen. Images licensed from Mai A Zhen. Uploaded by https://pan.baidu.com/s/1XH6-KGnSjfmtjhcMLLK3JQ?pwd=rbdx (accessed on 5 November 2025). Used under license. All rights reserved. These images are excluded from the article’s Creative Commons license and are used under license from Mai A Zhen.

Prior to conducting the questionnaire survey, inquiries were made to ascertain respondents’ prior knowledge of brands utilizing AR technology and their willingness to dedicate time to completing the survey. In the event of an affirmative response, subjects were obliged to partake in the survey, with researchers providing guidance throughout the process. First, in a relatively quiet experimental environment, participants were instructed to maintain a seated or upright posture and directly view the Changsha ICH brand augmented reality (AR) advertisement via a mobile device (Samsung Tab S8). The subjects were exposed to the Changsha ICH brand and product information through an augmented reality (AR) advertisement. Subsequently, participants were required to respond to questions based on their genuine feelings after viewing the content (as shown in Figure 3).

After the survey’s conclusion, a total of 350 questionnaires were collected. Following the screening process, three categories of invalid data were identified and subsequently excluded from the analysis. The categories encompassed data from respondents who did not complete the questionnaire, respondents who completed the questionnaire within one minute, and respondents who selected the same option for all questions prior to submitting the questionnaire. Following a thorough examination of the data, 305 questionnaires and data sets were deemed valid for analysis, yielding a validity rate of 87.1%.

## 5. Results

### 5.1. Sample Analysis

As shown in Table 4, in the total sample, 107 subjects identified as male and 198 subjects identified as female, constituting 35.1% and 64.9% of the total population, respectively. The demographic composition of the survey sample was as follows: 58.7% of respondents were aged 18 to 25, while those aged 26 to 30 constituted 26.2%, with the age range predominantly concentrated between 18 and 30. The data indicate that 68.2% of respondents were from Hunan Province and its surrounding regions in China, while 59.3% resided in urban areas. It is noteworthy that when queried about their exposure to or learning experiences with AR-related technologies or brand advertisements, 62% of respondents reported having either directly engaged with or been exposed to AR, indicating a relatively high level of familiarity with the technology. Additionally, 32% of respondents found AR ads interesting, novel, and enjoyable.

### 5.2. Reliability and Validity Testing

This study employed a questionnaire survey method to collect data, necessitating an assessment of measurement quality to ensure meaningful subsequent analysis. The data validation process was conducted using IBM SPSS 27.0 software, with the analysis being based on 305 valid questionnaires. Initially, the internal consistency of each dimension was analyzed via Cronbach’s alpha reliability testing. Cronbach’s alpha, a measure of internal consistency, ranges from 0 to 1. Values within this range are indicative of passing reliability tests, with higher values corresponding to greater reliability. In general, reliability coefficients below 0.6 are considered unreliable, those between 0.6 and 0.7 are reliable, those between 0.7 and 0.8 are relatively reliable, those between 0.8 and 0.9 are very reliable, and those between 0.9 and 1 are highly reliable. As shown in Table 5, all reliability values in this test result exceed 0.7, indicating that the reliability of each test indicator is relatively good.

The structural equation model was employed to assess the compatibility between the 305 questionnaire data obtained from the survey and the theoretical model of this study. The fit indices of the primary indicators were analyzed using AMOS 26.0 software. As shown in Table 6, the model fit results indicate: CMIN/DF = 2.453, falling within the excellent range of 1–3; RMSEA = 0.069, classified as good; IFI = 0.901 and CFI = 0.900, both excellent; TLI = 0.885, good. Consequently, the comprehensive analysis indicates that the proposed model demonstrates a satisfactory degree of fit.

In view of the fact that the CFA model demonstrates good fit, the average variance extracted (AVE) and composite reliability (CR) for each dimension within the scale are examined further. The verification process is outlined as follows: First, the standardized factor loadings (TSTIMATE) for each measurement item on its corresponding dimension must be calculated using the established CFA model. Subsequently, the AVE and CR values are to be computed using the respective formulas. Previous studies have discussed the criteria for judging average variance extracted (AVE) and Cronbach’s alpha coefficient (Cheung et al., 2023; Sijtsma & Pfadt, 2021). According to the standards, the Average Variance Extracted (AVE) must be at least 0.4, and the Composite Reliability (CR) must be at least 0.7 in order to indicate good convergent validity and composite reliability.

The calculation Formula (1) is as follows:

(1) $$\$lAVE\$=\frac{\left(\sum \lambda^{2}\right)}{N}$$

The calculation Formula (2) is as follows:

(2) $$lCR=\frac{(\sum \lambda {)}^{2}}{(\sum \lambda {)}^{2}+\sum \varepsilon }$$

Based on the analysis results in Table 7, it can be concluded that in this validity test, the AVE values for each dimension all exceeded 0.4, and the CR values all exceeded 0.7. Therefore, it can be demonstrated that each dimension exhibits good convergent validity and composite reliability.

According to the HTMT analysis method proposed by Henseler et al. (2015) in structural equation modelling, HTMT values below 0.9 are considered good, and values below 0.85 are considered excellent. As shown in the HTMT analysis results in Table 8.

**Figure 4:**
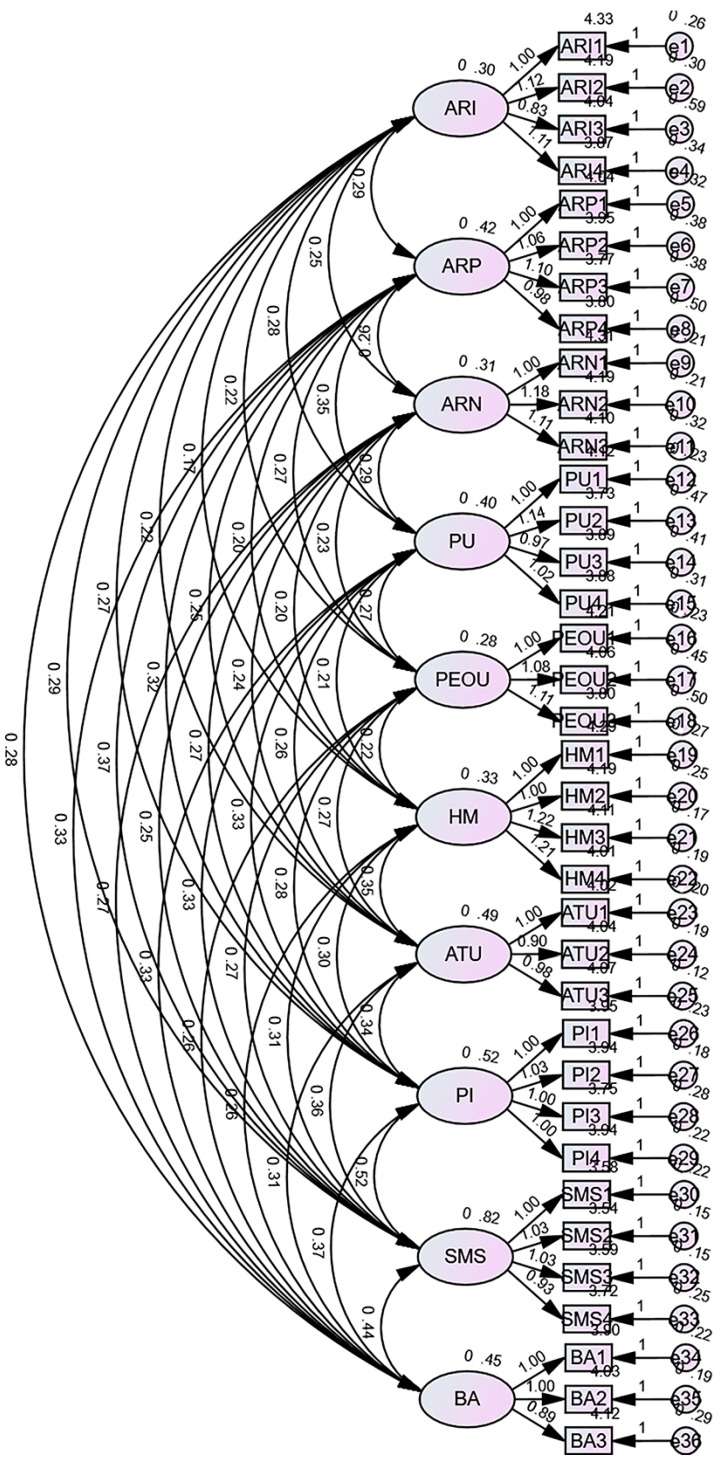
Confirmatory factor analysis (CFA) model. Source: The Figure was created by the author based on the survey results.

All HTMT values are below 0.85, indicating that the discriminant validity between the dimensions of the scale is excellent. The theoretical model of this study comprises ten measurement dimensions: AR interactivity, AR presence, AR novelty, perceived usefulness, perceived ease of use, hedonic motivation, attitude toward using, purchase intention, social media sharing, and brand attitude. To conduct confirmatory factor analysis on the scales, a confirmatory factor analysis model was constructed using AMOS, as shown in Figure 4, and computational results were generated.

### 5.3. Descriptive Statistics and Normality Tests

Table 9 presents the results of the normality test conducted in this study. Based on the statistical results of the descriptive analysis, it can be observed that the mean scores of all variables range from 3.32 to 4.21. The scoring method for the scale is a 1–5 positive scoring system. This indicates that the participants in this study have an above-average level of understanding regarding AR. The normality tests for each measurement item were conducted using skewness and kurtosis. According to the criteria proposed by Kline (1998), if the absolute value of the skewness coefficient is within 3 and the absolute value of the kurtosis coefficient is within 8, the data can be considered meeting the requirements of an approximate normal distribution. Based on the analysis results in Table 8, it can be seen that the absolute values of the skewness and kurtosis coefficients for all measurement items in this study are within the standard range.

In this analysis, Pearson correlation analysis was employed to explore the relationships among the variables. The findings suggest a substantial interconnection among the variables, indicating their potential for synergistic effects. Furthermore, these correlations are statistically significant at the 99% confidence level. As demonstrated in Table 10, the correlation coefficients r between all variables are greater than zero. These collective findings suggest that all variables in this analysis exhibit significant positive correlations.

### 5.4. Structural Equation Modeling and Hypothesis Testing

Preliminary analysis of the model fit indices in Table 11 indicates that the CMIN/DF (chi-square to degrees of freedom ratio) = 2.67 falls within the excellent range of 1–3. The RMSEA (root mean square error of approximation) = 0.074 also indicates a good fit. Furthermore, the IFI = 0.881, TLI = 0.868, and CFI = 0.88 all indicate a good fit. Consequently, the model in this study exhibits an adequate fit, as evidenced by the comprehensive analysis results.

The results of the path relationship test for the theoretical model are presented in Table 12. In this table, the path coefficient between two latent variables is represented by the estimate, thereby facilitating a comparison of their relative influence. The data reveal that AR presence exhibits positive path coefficients for all three latent variables—perceived usefulness, perceived ease of use, and hedonic motivation—with all results significant at the *p* < 0.05 level. Consequently, research hypotheses H2a, H2b, and H2c are supported by the survey data. Concurrently, AR novelty demonstrates positive path coefficients for all three latent variables—perceived usefulness, perceived ease of use, and hedonic motivation—with all correlations exhibiting significance at the *p* < 0.001 level. This finding suggests that the survey data support research hypotheses H3a, H3b, and H3c.

Furthermore, the path coefficients from perceived usefulness, perceived ease of use, and hedonic motivation (HM) to attitude toward using are all positive and significant at the *p* < 0.05 level, indicating that the survey data support research hypotheses H4, H5, and H6. Concurrently, the path coefficients from attitude toward using to purchase intention, Social media sharing, and brand attitude were all positive and highly significant at the *p* < 0.001 level, thereby substantiating the research hypotheses H7, H8, and H9. Finally, the path coefficients from social media sharing to purchase intention and brand attitude were both positive and highly significant at the *p* < 0.001 level, thus validating hypotheses H10 and H11.

## 6. Discussion

This study employs a combined theoretical model of TAM and UTAUT2 to examine how the AR attributes of ICH brands influence consumer attitudes and purchase intentions through emotional factors (hedonic motivation) and cognitive responses (perceived usefulness, perceived ease of use). The findings are explained below.

Initially, it is imperative to examine the factors that influence consumer attitudes toward the utilization and acquisition of ICH brands that incorporate AR technology. These factors encompass interactivity, presence, novelty, perceived usefulness, perceived ease of use, and hedonic motivation. The findings of the present study demonstrate that the presence and novelty of AR positively influence consumers’ attitudes toward using through perceived usefulness, perceived ease of use, and hedonic motivation. Furthermore, consumers’ positive attitudes toward AR usage positively influenced their purchase intentions for ICH brands, consistent with the rationale demonstrated in Fan et al. (2022) for integrating AR characteristic dimensions with the TAM model. The introduction of interactivity, presence, and novelty as AR attributes in this study expands upon the dimensions of AR attributes for ICH brands proposed in Li et al. (2022).

Secondly, the study found that among the various attributes of AR, novelty in ICH brand AR technology exerted the most significant influence on consumers’ perceived usefulness, perceived ease of use, and hedonic motivation. However, Nikhashemi et al. (2021) emphasized that the novelty of AR has a greater impact on consumers compared to other attributes, and the findings of this study are mainly consistent with theirs. Consequently, the primary emphasis for ICH brand operators and AR technology developers should be on the effective integration of creative elements and novel experiences into AR environments, thereby inducing pleasurable experiences for consumers through these innovative AR settings. Despite the documented positive influence of presence and novelty in AR experiences on users’ perceived usefulness, perceived ease of use, and hedonic motivation, the present study’s findings do not demonstrate a positive impact of interactivity in intangible cultural heritage brand AR experiences on consumers’ perceived usefulness, perceived ease of use, and hedonic motivation. This finding stands in contrast to the research results reported by Arghashi and Yuksel (2022). The specific reasons for the discrepancies in these research findings lie, on the one hand, in the scenario construction associated with the interaction between ICH brands and AR. Consumers may be unable to directly and effectively comprehend the expressive intent of AR designers, leading to cognitive differences during technological interactions. On the other hand, the interactive functions of AR in interface design may fail to meet the diverse exploratory needs of different consumer groups.

Thirdly, within the TAM framework, the findings indicate that consumers’ perceived ease of use exerts a significant positive influence on their attitude toward using. This finding aligns with the original theoretical foundation of TAM proposed by Davis (1989), indicating that when consumers perceive a technological product as easy to operate, their willingness to adopt and use it is markedly enhanced. Therefore, it is essential to enhance the knowledge content, intangible cultural value, and practicality of AR technology, enrich its historical and cultural significance, and strengthen its educational and popular science dissemination functions to ensure it meets consumers’ real-world needs. Conversely, consumers who hold a favorable attitude toward AR systems exhibit increased behavioral intentions, thereby enhancing their propensity to utilize such systems. Moreover, this study demonstrates that perceived value has the capacity to elicit psychological responses in consumers. Specifically, the hedonic motivation within consumers’ perceived value, similar to perceived ease of use, exerts a positive influence on attitude toward using. When consumers encounter pleasure during an AR experience, they develop a favorable attitude toward the AR technology, thereby increasing their propensity to adopt it.

Fourth, within the ICH brand AR context, this study confirms that consumers’ attitudes toward using AR technology positively influence their purchase intention, social media sharing, and brand attitude. Simultaneously, social media sharing positively impacts consumers’ purchase intention and brand attitude. Specifically, when consumers demonstrate a positive attitude toward using AR technology, they are more likely to purchase ICH brands featuring AR technology and their associated products. They are also more inclined to share this unique ICH brand AR experience and their impressions with friends or family via social media. This sharing sparks curiosity and attention toward the ICH brand and its products, fostering positive brand attitudes. Moreover, social media sharing has enhanced the brand’s visibility and influence to a certain extent, while also stimulating consumers’ purchase intent. In summary, AR technology has enriched the consumer experience, with positive user experiences yielding the following impacts: promoting social media sharing, boosting purchase intent through improved brand sentiment, and enabling brand interaction via AR technology (Sung et al., 2022).

Fifth, these study findings hold practical significance and implications for the application of AR technology in ICH brand building and marketing. From a tool perspective, AR technology can enhance consumer experiences. Typically, AR serves as a marketing tool in the early stages to promote brands. In the mid-term, it functions as an interactive tool to elevate consumer perceptions. In the later stages, AR is also regarded as a means to shape positive brand images and reputation.

Sixth, this study holds reference value for AR designers within ICH brands. AR design for ICH brands must fully embody the attributes of presence and novelty. First, AR visual design should maintain consistency with the brand’s style, enhancing information while ensuring the authenticity of three-dimensional scene creation. Emphasis should be placed on the vividness of visual imagery and the adaptability of sound effects’ integration to enhance consumers’ sense of presence. Second, streamlined, intuitive operational workflows should reduce visual fatigue and operational complexity, while personalized interactive features cater to diverse consumer needs across different segments.

Furthermore, ICH brand AR design must continuously discover and create novel visual elements. This process involves deeply exploring cultural history and blending traditional heritage with contemporary pop culture or visual motifs to form universally appealing visual forms, thereby enhancing AR novelty. Finally, ICH brand operators should prioritize leveraging the dissemination capabilities of social media platforms. Encouraging consumers to share their AR brand experiences and positive impressions on social media amplifies brand influence and recognition, stimulates mass consumption, and generates both economic value and socio-cultural value.

Consequently, the findings of this study also demonstrate the significant value of AR technology for the sustainable development of ICH brands. It not only enhances the appeal and dissemination of ICH brands but also promotes the deep integration of their commercial and social value. In the preservation and transmission of ICH, innovative approaches such as AR—which are digital, interactive, and contextual—address core challenges in traditional cultural preservation, including incomplete documentation, insufficient dissemination, and a shortage of practitioners. This technology provides a sustainable pathway for the transmission of ICH.

This study has several limitations. By focusing solely on the ICH brand in Changsha, Hunan Province, China, this regionally confined research may have inherent limitations. Future studies could prioritize examining ICH brands across different countries or regions worldwide to validate the model’s universality. Second, this study did not compare differences in AR attitudes toward using ICH brands across distinct age groups. The majority of the sample comprised young adults aged 20 to 30, who may exhibit a predisposition toward embracing new technologies. Consequently, future research should consider expanding the survey sample to include diverse age cohorts, examining and contrasting usage attitudes and consumption characteristics among different age groups.

## 7. Conclusions, Implications, and Future Research Directions

This study innovatively integrates the TAM and UTAUT2 theoretical frameworks to construct a research model examining the impact of introducing AR experiences in ICH brands on purchase intention, while exploring the interrelationships among various factors. Within the ICH brand, AR serves as an effective tool and medium. Through its inherent attributes—presence and novelty—it enhances consumers’ emotional factors (hedonic motivation) and cognitive responses (perceived usefulness, perceived ease of use). This transformation fosters positive brand attitudes and purchasing behavior, thereby driving consumption. Specifically, the presence and novelty inherent in AR media directly stimulate consumers, eliciting pleasurable emotional responses and psychological experiences. These positive reactions carry strong social attributes. Driven by the desire to showcase novelty and share joy, consumers post their AR experiences with ICH brands on social media. This practice helps build a positive ICH brand image, bolsters the brand’s marketing power and communication reach, and ultimately expands its overall influence. Therefore, when designing AR products for the ICH brand, designers should prioritize creating immersive historical and cultural scenarios for consumers. By blending the rich local heritage with contemporary popular culture, they can craft novel AR visual experiences that deliver genuine enjoyment to consumers.

### 7.1. Theoretical Implications

This study fills a theoretical gap at the intersection of digital technology, emotional marketing, and cultural heritage consumption. First, it theoretically integrates and extends two classic technology adoption models—TAM and UTAUT2—within an innovation context. TAM provides a solid foundation for understanding consumer acceptance of technological systems, while UTAUT2 introduces key consumer value dimensions such as hedonic motivation and price value, which are particularly crucial in discretionary consumption scenarios. This integrated model demonstrates enhanced explanatory power when interpreting consumer behavior in cultural heritage experiences, proving that a hybrid framework better captures the complexity of technology-enabled cultural consumption than any single model. Previous researchers have noted that when consumers perceive a brand’s emotional commitment as lacking authenticity, they tend to avoid that brand (Thompson et al., 2006). By proactively integrating the theoretical frameworks of emotional branding and AR experiential marketing, this study moves beyond the traditional focus on technology adoption models and consumers’ perceptions of brand emotional authenticity. Instead, it highlights the transformation of AR technology from a rational tool into a medium for creating brand emotional value, thereby extending theoretical discussions from the dimension of functional utility to that of emotional experience. In doing so, the study transcends conventional technology adoption models and their associated theoretical boundaries.

Second, this study introduces and empirically validates emotional factors and cognitive responses as core mediating mechanisms. Previous technology adoption research has often focused on cognitive and utilitarian pathways. By positioning consumer emotional factors and cognitive responses as key mediators between AR technology attributes and consumer attitudes/behavioral outcomes, this study achieves a paradigm shift. It emphasizes that within intangible cultural heritage brand contexts, AR technology’s value lies not only in practicality but also in its capacity to “technologically enable emotion”—this emotional bond serves both as a prerequisite for commercial transactions and as the cornerstone for expanding brand dissemination.

Third, this study positions AR research within the understudied realm of intangible cultural heritage. While past AR studies have predominantly focused on retail, gaming, or tangible goods, its application to intangible cultural heritage brands fills a significant gap, revealing how advanced technology can be leveraged to protect and revitalize fragile cultural assets. This research provides a theoretical blueprint for understanding how to make abstract, skill-based cultural heritage more accessible, relatable, and commercially viable to contemporary audiences.

### 7.2. Practical Implications

This study’s findings provide guidance for AR design in ICH brands and offer practical reference value for developers designing and building AR system platforms for ICH. When designing AR products for intangible cultural heritage brands, designers should prioritize creating immersive historical and cultural scenarios for consumers. Building upon authentic reconstructions of cultural heritage, they must emphasize seamless integration between AR visual effects and real-world environments, achieving perfect fusion between the virtual and the real. Simultaneously, designers should blend deep-rooted local history and culture with contemporary popular culture to create novel AR visual experiences that delight users. When designing AR products related to ICH, designers should avoid creating “technological hollow shells.” Instead of merely piling on special effects, they should integrate AR technology with the spiritual essence of cultural heritage. Furthermore, they should fully consider the varying needs and operational difficulties different audiences face regarding technological interactivity. Adopting interface designs that are easy to operate, interact with, and understand will help reach a broad consumer base.

This study’s findings hold significant value and contribution in revealing the impact of AR experiences on the sustainable development of ICH brands. AR provides revolutionary tools for ICH brands, effectively addressing the communication challenges posed by the intangible nature of cultural heritage through immersive experiences, interactive dissemination, and the revitalization of cultural values. AR technology drives consumer engagement and conversion within ICH brands. By sharing their AR experiences on social media and platforms, consumers create a viral social dissemination and business model. This significantly advances the sustainable development and popularization of intangible cultural heritage and its associated brands, overcoming the constraints of geographical limitations and narrow commercial pathways traditionally faced by ICH.

Furthermore, this study contributes to understanding the formation mechanism of user adoption intent for AR products within the ICH brand, offering practical reference value and real-world significance for ICH brand development and AR marketing. AR technology represents a novel approach for industrial development and product transformation of ICH resources, serving as an effective means to enhance the visibility and influence of ICH brands. Through digital empowerment, immersive experiences, and the reconstruction of commercial value, AR achieves a win-win scenario for cultural heritage preservation and consumption growth. It injects new vitality into promoting more sustainable ICH protection and dissemination, as well as the development of the digital creative industry.

### 7.3. Future Research Direction

The primary limitation of this study lies in the geographical and cultural specificity of the sample. Data were collected exclusively from consumers in Changsha City, Hunan Province, China. As a dynamic modern provincial capital, Changsha’s consumer population exhibits distinct demographic characteristics (e.g., educational attainment, income, technological proficiency) and a cultural background influenced by both local Hunan traditions and national trends. Consequently, the generalizability (external validity) of this study’s model may be constrained. The correlations identified among variables—particularly the strong association between hedonic motivation and perceived price value—may differ across cultural contexts (e.g., more conservative or less developed regions and countries), levels of economic development, or areas with varying exposure to ICH. Additionally, the majority of the study sample comprised young adults aged 20 to 30, who may exhibit a propensity toward embracing new technologies. Given these limitations, this paper proposes several promising directions for future research. To address geographical limitations, future research should adopt cross-cultural and cross-national comparative designs. For instance, replicating this study in other Chinese cities with deep ICH traditions but distinct cultural characteristics (e.g., Xi’an, Suzhou, or Lhasa) could examine intra-cultural variations. A more forward-looking approach would involve cross-national comparisons among consumers from Eastern and Western cultures (e.g., China, Japan, France, and the United States), which would yield highly insightful findings. Furthermore, building on the findings of this study, subsequent research could explore the role of individual difference variables as moderators. Factors such as consumers’ cultural identity, nostalgia tendencies, technophobia, and innovativeness may significantly influence their responses to AR experiences. For instance, compared to consumers with a globalized identity, AR technology may evoke stronger emotional impacts among those with a strong sense of local cultural identity.

## Figures and Tables

**Table 1:** The history of AR technology development.

Period	Key Technological Development	Marketing Integration Example
1950–1990	Foundational research (Sensorama, HMDs)	None
1990–2005	Early AR systems	Experimental corporate uses
2005–2012	Mobile AR, marker-based	Pepsi, Starbucks AR campaigns
2013–2016	Google Glass, Pokémon GO	Gamified brand engagement
2017–2020	AR Kit, AR Core, social filters	Snapchat/Instagram brand filters

Source: The table adapted from Sünger and Çankaya (2019).

**Table 2:** Changsha’s representative ICH brands.

ICH Brand Name	Category	Brand Images	Area
Fire God Palace (a)	Traditional snack-making skills	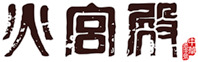	Changsha Pozi Street
Jiu Zhi Tang (b)	Chinese medicine preparation	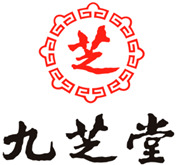	Changsha Taiping Street
Yu Lou Dong (c)	Noodle-making skills	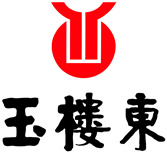	Changsha Beizheng Street

Source: The table was compiled and created by the author. Figure (a). Fire God Palace logo © Shizilukou. Images licensed from Sj51 (www.sj51.net). Used under license. All rights reserved. These images are excluded from the article’s Creative Commons license and are used under license from Sj51; Figure (b). Jiu Zhi Tang logo © jcc881120. Images licensed from Pngsucai (www.pngsucai.com). Used under license. All rights reserved. These images are excluded from the article’s Creative Commons license and are used under license from Pngsucai; Figure (c). Yu Lou Dong logo © Xinyuloudong. Images licensed from Baidu (www.baidu.com). Used under license. All rights reserved. These images are excluded from the article’s Creative Commons license and are used under license from Baidu.

**Table 3:** Scale variables and item settings.

Variable	Index	Content	Source
Interactivity	ARI1	I prefer to use Changsha Intangible Cultural Heritage Brand Augmented Reality Advertising which can effectively collect user feedback.	(Arghashi & Yuksel, 2022; Nikhashemi et al., 2021)
	ARI2	I prefer watching the Changsha Intangible Cultural Heritage brand advertisement using augmented reality technology because it makes me feel like it wants to listen to me.	
	ARI3	I have some control over what I want to watch.	
	ARI4	The augmented reality advertising of Changsha’s intangible cultural heritage brand can quickly respond to my specific needs.	
Presence	ARP1	While experiencing the augmented reality ads, I felt like I was in the virtual world they created.	(Sung et al., 2022)
	ARP2	While experiencing augmented reality ads, my body was in the real world, but my mind was in the virtual world it created.	
	ARP3	When I experienced augmented reality ads, I often thought I was actually in the physical location.	
	ARP4	When I experience augmented reality ads, I feel like my mind is in the actual location.	
Novelty	ARN1	Augmented reality advertising can offer something new.	(Nikhashemi et al., 2021)
	ARN2	Watching augmented reality ads provides unique intangible cultural heritage brand information.	
	ARN3	Augmented reality ads can provide special content.	
Perceived usefulness	PU1	These augmented reality ads have improved my ability to choose brands as well as intangible cultural heritage products more effectively.	(Arghashi & Yuksel, 2022; Recalde et al., 2024)
	PU2	I feel like these augmented reality ads will make my life easier.	
	PU3	I think watching augmented reality ads will make my shopping or consumption more efficient.	
	PU4	I feel like augmented reality ads will help me accomplish things I might need.	
Perceived ease of use	PEOU1	Watch the augmented reality ad, clear and easy to understand.	(Recalde et al., 2024)
	PEOU2	Watching an augmented reality ad doesn’t require much brainpower.	
	PEOU3	It was easy to find the product I wanted by watching the augmented reality ads.	
Hedonic motivation	HM1	Watching the AR ad is fun.	(Schapsis et al., 2025; Sia et al., 2024)
	HM2	AR ad content makes shopping or consumption fun.	
	HM3	AR ads are enjoyable.	
	HM4	AR ads are exciting.	
Attitude of using	ATU1	This AR ad is good.	(Yim et al., 2017)
	ATU2	This AR ad is popular.	
	ATU3	I enjoy watching the AR ad.	
Purchase intention	PI1	I am willing to recommend others to buy Changsha Intangible Cultural Heritage products with AR ads.	(Hu et al., 2025; Recalde et al., 2024)
	PI2	In the future, I am very interested in buying Changsha Intangible Cultural Heritage brand products with AR ads.	
	PI3	In the future, the use of AR in Changsha Intangible Cultural Heritage brands is important to me.	
	PI4	Watching the AR ad encourages me to try the Intangible Cultural Heritage product in the near future.	
Social media sharing	SMS1	I want to post about this AR ad experience on social media.	(Sung et al., 2022)
	SMS2	I want to share this AR ad experience on my social media.	
	SMS3	I will recommend these AR ads to others on my social media.	
	SMS4	I want to tell others on my social media about the positive feelings about this AR experience.	
Brand attitude	BA1	After watching the Intangible Cultural Heritage ad with AR technology, it is a wise choice to use or choose this brand.	(Arghashi & Yuksel, 2022)
	BA2	If there is another brand that is just as good as the non-legacy brand, I will be more inclined to use the non-legacy brand because I have experienced it through AR.	
	BA3	If there is no difference between the other brand and the non-legacy brand, it seems more sensible to use the non-legacy brand because I know it through AR.	

Source: The table was created by the author based on the survey results.

**Table 4:** Demographic characteristics.

Variable	Options	Frequency	Percentage
Gender	Male	107	35.1
	Female	198	64.9
Age	Under 18	4	1.3
	18–25	179	58.7
	26–30	80	26.2
	31–40	37	12.1
	41–50	3	1
	51–60	2	0.7
location	Hunan	208	68.2
	Other	97	31.8
Place of residence	Country	124	40.7
	City	181	59.3
AR experience	Yes	189	62
	No	116	38

Source: The table was created by the author based on the survey results.

**Table 5:** Reliability analysis.

Variable	Cronbach Alpha	Number of Items
ARI	0.768	4
ARP	0.819	4
ARN	0.816	3
PU	0.835	4
PEOU	0.713	3
HM	0.882	4
ATU	0.889	3
PI	0.902	4
SMS	0.943	4
BA	0.841	3
Total	0.958	41

Source: The table was created by the author based on the survey results.

**Table 6:** Model fitness test.

Index	Reference Standards	Result
CMIN/DF	Greater than 1 and less than 3 is excellent; greater than 3 and less than 5 is good	2.453
RMSEA	Less than 0.05 is excellent; less than 0.08 is good	0.069
IFI	Greater than 0.9 is excellent; greater than 0.8 is good	0.901
TLI	Greater than 0.9 is excellent; greater than 0.8 is good	0.885
CFI	Greater than 0.9 is excellent; greater than 0.8 is good	0.9

Source: The table was created by the author based on the survey results.

**Table 7:** Convergent validity and combined reliability tests of each dimension.

Path Relationship			Estimate	AVE	CR
ARI1	←	ARI	0.732	0.471	0.777
ARI2	←	ARI	0.752		
ARI3	←	ARI	0.511		
ARI4	←	ARI	0.723		
ARN1	←	ARN	0.768	0.598	0.817
ARN2	←	ARN	0.814		
ARN3	←	ARN	0.736		
ARP1	←	ARP	0.753	0.535	0.821
ARP2	←	ARP	0.745		
ARP3	←	ARP	0.757		
ARP4	←	ARP	0.668		
PU1	←	PU	0.797	0.555	0.833
PU2	←	PU	0.728		
PU3	←	PU	0.693		
PU4	←	PU	0.759		
PEOU1	←	PEOU	0.739	0.458	0.716
PEOU2	←	PEOU	0.65		
PEOU3	←	PEOU	0.637		
HM1	←	HM	0.747	0.648	0.88
HM2	←	HM	0.755		
HM3	←	HM	0.863		
HM4	←	HM	0.848		
ATU1	←	ATU	0.846	0.732	0.891
ATU2	←	ATU	0.825		
ATU3	←	ATU	0.895		
SMS1	←	SMS	0.888	0.808	0.944
SMS2	←	SMS	0.925		
SMS3	←	SMS	0.922		
SMS4	←	SMS	0.858		
PI1	←	PI	0.833	0.67	0.903
PI2	←	PI	0.87		
PI3	←	PI	0.807		
PI4	←	PI	0.835		
BA1	←	BA	0.822	0.645	0.845
BA2	←	BA	0.839		
BA3	←	BA	0.745		

Source: The table was created by the author based on the survey results.

**Table 8:** Discriminant validity test.

Variable	ARI	ARN	ARP	PU	PEOU	HM	ATU	SMS	PI	BA
ARI										
ARN	0.817									
ARP	0.816	0.727								
PU	0.783	0.794	0.846							
PEOU	0.749	0.788	0.77	0.796						
HM	0.556	0.658	0.544	0.59	0.741					
ATU	0.571	0.637	0.561	0.602	0.729	0.837				
SMS	0.599	0.523	0.649	0.572	0.592	0.602	0.577			
PI	0.682	0.669	0.688	0.716	0.732	0.719	0.671	0.824		
BA	0.752	0.727	0.764	0.757	0.749	0.676	0.661	0.734	0.784	

Source: The table was created by the author based on the survey results.

**Table 9:** Normality test.

Variable	Mean Value	Standard Deviation	Skewness	Kurtosis
ARI1	4.33	0.755	−1.133	1.762
ARI2	4.19	0.826	−0.972	0.943
ARI3	4.04	0.897	−0.787	0.348
ARI4	3.87	0.848	−0.462	0.162
ARP1	4.04	0.859	−0.834	0.576
ARP2	3.95	0.923	−0.905	0.823
ARP3	3.77	0.942	−0.477	−0.301
ARP4	3.8	0.952	−0.597	−0.001
ARN1	4.31	0.72	−1.191	2.606
ARN2	4.19	0.8	−1.049	1.674
ARN3	4.1	0.836	−0.743	0.378
PU1	4.12	0.798	−0.957	1.523
PU2	3.73	0.997	−0.664	0.201
PU3	3.89	0.895	−0.701	0.404
PU4	3.88	0.852	−0.569	0.324
PEOU1	4.21	0.716	−0.814	1.241
PEOU2	4.06	0.881	−1.023	1.164
PEOU3	3.8	0.922	−0.539	−0.009
HM1	4.29	0.776	−1.069	1.425
HM2	4.19	0.767	−1.086	2.193
HM3	4.11	0.821	−0.933	1.223
HM4	4.01	0.827	−0.586	0.219
ATU1	4.02	0.833	−0.834	1.125
ATU2	4.04	0.771	−0.669	0.858
ATU3	4.07	0.77	−0.82	1.476
PI1	3.95	0.865	−0.71	0.471
PI2	3.94	0.855	−0.657	0.437
PI3	3.75	0.894	−0.267	−0.296
PI4	3.94	0.862	−0.791	0.809
SMS1	3.58	1.02	−0.36	−0.349
SMS2	3.54	1.006	−0.386	−0.151
SMS3	3.59	1.009	−0.48	−0.084
SMS4	3.72	0.976	−0.627	0.203
BA1	3.9	0.821	−0.641	0.848
BA2	4.03	0.802	−0.867	1.509
BA3	4.12	0.811	−1.002	1.534

Source: The table was created by the author based on the survey results.

**Table 10:** Pearson correlation analysis between various dimensions.

Dimensions	ARI -AVE	ARP -AVE	ARN -AVE	PU -AVE	PEOU -AVE	HM -AVE	ATU -AVE	PI -AVE	SMS -AVE	BA -AVE
**ARI** **-AVE**	1									
**ARP** **-AVE**	0.647 **	1								
**ARN** **-AVE**	0.648 **	0.594 **	1							
**PU** **-AVE**	0.624 **	0.697 **	0.650 **	1						
**PEOU** **-AVE**	0.552 **	0.583 **	0.597 **	0.607 **	1					
**HM** **-AVE**	0.456 **	0.462 **	0.557 **	0.502 **	0.588 **	1				
**ATU** **-AVE**	0.473 **	0.479 **	0.546 **	0.520 **	0.579 **	0.745 **	1			
**PI** **-AVE**	0.566 **	0.590 **	0.576 **	0.617 **	0.590 **	0.641 **	0.600 **	1		
**SMS** **-AVE**	0.511 **	0.570 **	0.460 **	0.504 **	0.493 **	0.548 **	0.528 **	0.760 **	1	
**BA** **-AVE**	0.603 **	0.633 **	0.601 **	0.629 **	0.577 **	0.582 **	0.572 **	0.684 **	0.653 **	1

Source: The table was created by the author based on the survey results. ** At the 0.01 level (double-tailed), the correlation is significant.

**Table 11:** Goodness-of -fit test of the structural equation model.

Index	Reference Standards	Result
CMIN/DF	Greater than 1 and less than 3 is excellent; greater than 3 and less than 5 is good	2.67
RMSEA	Less than 0.05 is excellent; less than 0.08 is good	0.074
IFI	Greater than 0.9 is excellent; greater than 0.8 is good	0.881
TLI	Greater than 0.9 is excellent; greater than 0.8 is good	0.868
CFI	Greater than 0.9 is excellent; greater than 0.8 is good	0.88

Source: The table was created by the author based on the survey results.

**Table 12:** Path relationship test results.

				Estimate	S.E.	C.R.	*p*	Result
H1	PU	←	ARI	−0.036	0.144	−0.257	0.797	Reject
H2	PEOU	←	ARI	−0.201	0.154	−1.14	0.254	Reject
H3	HM	←	ARI	−0.245	0.207	−1.36	0.174	Reject
H4	PU	←	ARP	0.541	0.113	4.802	***	Accept
H5	PEOU	←	ARP	0.505	0.114	3.797	***	Accept
H6	HM	←	ARP	0.288	0.146	2.216	0.027	Accept
H7	PU	←	ARN	0.462	0.11	4.234	***	Accept
H8	PEOU	←	ARN	0.627	0.119	4.545	***	Accept
H9	HM	←	ARN	0.654	0.161	4.599	***	Accept
H10	ATU	←	PU	0.099	0.085	1.224	0.221	Reject
H11	ATU	←	PEOU	0.237	0.108	2.707	0.007	Accept
H12	ATU	←	HM	0.687	0.054	11.8	***	Accept
H13	SMS	←	ATU	0.624	0.078	10.946	***	Accept
H14	PI	←	ATU	0.408	0.058	7.636	***	Accept
H15	BA	←	ATU	0.457	0.065	7.366	***	Accept
H16	PI	←	SMS	0.552	0.044	10.05	***	Accept
H17	BA	←	SMS	0.453	0.046	7.477	***	Accept
H18	ARI3	←	ARI	0.518	0.091	8.357	***	Accept
H19	ARI2	←	ARI	0.748	0.085	11.95	***	Accept
H20	ARI1	←	ARI	0.731	0.077	11.692	***	Accept
H21	ARP3	←	ARP	0.753	0.102	11.11	***	Accept
H22	ARP2	←	ARP	0.743	0.1	10.983	***	Accept
H23	ARP1	←	ARP	0.759	0.093	11.175	***	Accept
H24	ARN2	←	ARN	0.792	0.076	13.385	***	Accept
H25	ARN1	←	ARN	0.763	0.069	12.883	***	Accept
H26	PU2	←	PU	0.739	0.088	13.287	***	Accept
H27	PU3	←	PU	0.705	0.08	12.579	***	Accept
H28	PU4	←	PU	0.756	0.075	13.647	***	Accept
H29	PEOU2	←	PEOU	0.653	0.103	10.437	***	Accept
H30	PEOU3	←	PEOU	0.629	0.108	10.06	***	Accept
H31	HM3	←	HM	0.864	0.054	18.752	***	Accept
H32	HM2	←	HM	0.752	0.054	15.193	***	Accept
H33	HM1	←	HM	0.744	0.055	14.968	***	Accept
H34	ATU2	←	ATU	0.811	0.054	17.434	***	Accept
H35	ATU1	←	ATU	0.824	0.058	17.916	***	Accept
H36	PI2	←	PI	0.872	0.055	18.69	***	Accept
H37	PI3	←	PI	0.812	0.06	16.779	***	Accept
H38	PI4	←	PI	0.828	0.057	17.263	***	Accept
H39	SMS2	←	SMS	0.922	0.041	24.849	***	Accept
H40	SMS3	←	SMS	0.923	0.041	24.925	***	Accept
H41	SMS4	←	SMS	0.86	0.044	21.264	***	Accept
H42	BA2	←	BA	0.834	0.059	16.316	***	Accept
H43	BA3	←	BA	0.718	0.062	13.515	***	Accept

Source: The table was created by the author based on the survey results. Note: *** indicate significance at the 0.1% level.

## Data Availability

The datasets used and/or analyzed during the current study are available from the corresponding author upon reasonable request.

## Abbreviations

The following abbreviations are used in this manuscript:

ICH	Intangible Cultural Heritage
AR	Augmented Reality
UNESCO	United Nations Educational, Scientific, and Cultural Organization
GPS	Global Positioning System
SDG	Sustainable Development Goals
TAM	Technology Acceptance Model
UTAUT2	Unified Theory of Acceptance and Use of Technology 2
